# Triglyceride-glucose index is associated with a higher risk of stroke in a hypertensive population

**DOI:** 10.1186/s12933-023-02082-1

**Published:** 2023-12-13

**Authors:** Nan Zhang, Xiying Chi, Ziyi Zhou, Yun Song, Shuqun Li, Jiafeng Xu, Jianping Li

**Affiliations:** 1 Department of Cardiology, Peking University First Hospital, No. 8 Xishiku St, Xicheng District, Beijing, 100034 China; 2 Tsinghua Shenzhen International Graduate School, Tsinghua University, Shenzhen, China; 3 Shenzhen Evergreen Medical Institute, Shenzhen, China; 4 Department of Gastrointestinal Surgery, Beijing Shijitan Hospital, Capital Medical University, Beijing, China; 5 Department of Epidemiology, School of Public Health, Southern Medical University, Guangzhou, Guangdong China

**Keywords:** Triglyceride-glucose index, Stroke, Age

## Abstract

**Background:**

This study aimed to evaluate the association of triglyceride-glucose (TyG) index, an insulin resistance surrogate biomarker, with first stroke in a hypertensive population and to explore potential influencing factors.

**Methods:**

This study, a cohort study among a rural Chinese hypertensive population, utilized data from the China Stroke Primary Prevention Trial (CSPPT). The TyG index was calculated as ln [fasting triglyceride (mg/dL) × fasting glucose (mg/dL)/2]. Multivariate analysis using Cox proportional hazards models was conducted.

**Results:**

A total of 7569 hypertensive patients were included in this study. When TyG index was assessed as quartiles, compared with the reference group (Quartile 1), the hazard ratio of stroke was 1.04 in Quartile 2, 1.43 in Quartile 3, and 1.45 in Quartile 4, with a significant trend test (*P* = 0.013). When Quartiles 3 and 4 were combined (≥ 8.8), the hazard ratio was 1.41 compared with combined Quartiles 1 and 2 (< 8.8). Similar findings were observed for the association of TyG index with ischemic stroke. Further, a joint effect of baseline TyG index and age on first stroke was found. Using the group with TyG < 8.8 and age < 60 years as a reference, the highest hazard ratio of stroke was found in the group with a higher TyG index and aged 60 or greater (HR: 2.15, 95% CI 1.50, 3.07, P < 0.001).

**Conclusions:**

In a hypertensive population, baseline TyG index was associated with a significantly higher risk of first stroke. In addition, age was a significant effect modifier for this association.

**Supplementary Information:**

The online version contains supplementary material available at 10.1186/s12933-023-02082-1.

## Introduction

Insulin resistance (IR) is a common risk factor for stroke, and is also considered to be an important marker for other stroke risk factors such as diabetes and dyslipidemia, driving the onset and progression of stroke [1, 2]. The triglyceride-glucose (TyG) index, calculated from ln [fasting triglycerides (mg/dL) × fasting glucose (mg/dL)/2] [3], is considered to be a reliable and convenient alternative to IR with higher sensitivity and specificity compared to the IR gold standard [4]. Evidence suggests that the TyG index holds promise for greater value in the field of stroke prevention [5]. As a reliable IR surrogate biomarker that is easily obtained by blood tests and simple calculations, clarifying the predictive value of the TyG index and its possible influence on stroke is of great clinical and public health significance.

To date, important knowledge gaps in the relationship between TyG index and stroke remain. First, although some studies have demonstrated that TyG index is associated with a higher risk of stroke [6, 7], no studies have validated the association between TyG index and stroke in a hypertensive population. As an important high-risk group for stroke, the continued development of validated predictors of patients with hypertension is warranted. Second, no study has yet explored in depth the influential factors that may affect the association between TyG index and stroke. This study, a post-hoc analysis of a cohort from the China Stroke Primary Prevention Trial (CSPPT), aimed to validate the association between baseline TyG index and stroke risk in a Chinese hypertensive population, and also to explore possible influencing factors.

## Materials and methods

### Study design and participants

This study was a post-hoc analysis of the CSPPT. The methodology and primary outcomes of the CSPPT have been previously reported [8]. In brief, the CSPPT was a randomized, double-blind, controlled clinical trial conducted in 32 communities in China from 19 May 2008 to 24 August 2013. The study included 20,702 patients with hypertension aged 45–75 years, with hypertension defined as seated, resting systolic blood pressure ≥ 140 mmHg or diastolic blood pressure ≥ 90 mmHg, or taking antihypertensive medication at the time of screening or recruitment visit. After stratification according to the methylenetetrahydrofolate reductase (MTHFR) C677T genotype (CC, CT or TT), patients were randomized in a 1:1 ratio to either the enalapril group (a daily oral dose of 1 tablet containing 10 mg of enalapril only) or the enalapril-folic acid group (a daily oral dose of 1 tablet containing 10 mg of enalapril and 0.8 mg of folic acid), with the primary endpoint of the study set at first stroke. Participants were followed up every three months and a total of 637 stroke events occurred during a median treatment period of 4.5 years. This trial was registered at clinicaltrials.gov as NCT00794885.

This study utilized the patient population within the enalapril group of the CSPPT to analyze the relationship between baseline TyG index and first stroke.  The initial sample consisted of 7947 patients with hypertension from the Lianyungang Center, and after excluding those with missing baseline triglyceride and glucose data, a total of 7569 participants were included in this study (Additional file 1: Fig. S1).

### Data availability statement and patient consent

This article adheres to the Strengthening the Reporting of Observational Studies in Epidemiology (STROBE) guideline [9]. The parent study was approved by the ethics committee of the Institute of Biomedicine, Anhui Medical University, Hefei, China (FWA assurance number FWA00001263). All participants provided written, informed consent. Data, analytical methods and research materials supporting the results of this study may be reasonably requested from the corresponding author after the application has been submitted and formally reviewed and approved by the Ethics Committee of the Institute of Biomedical Research, Anhui Medical University.

### Outcomes assessment

The primary outcome was a first nonfatal or fatal stroke, excluding subarachnoid hemorrhage and silent stroke. All study outcomes were reviewed and adjudicated according to standard criteria by an independent Endpoint Adjudication Committee.

### Laboratory assays

Baseline serum fasting glucose, fasting lipid and homocysteine levels were measured using automatic clinical analyzers (Beckman Coulter) at the core laboratory of the National Clinical Research Center for Kidney Disease, Nanfang Hospital, Guangzhou, China. Baseline serum folate and vitamin B12 were measured by a commercial laboratory using a chemiluminescent immunoassay (New Industrial). Estimated glomerular filtration rate was calculated using the Chronic Kidney Disease Epidemiology Collaboration (CKD-EPI) equation. MTHFR C677T polymorphisms were detected on an ABI Prism 7900HT sequence detection system (Life Technologies) using the TaqMan assay.

TyG index was calculated as ln [fasting triglyceride (mg/dL) × fasting glucose (mg/dL)/2].

### Statistical analysis

Means ± SD or medians (25th percentile–75th percentile) and proportions were calculated for population characteristics by median of TyG index. Multivariate analyses were performed using Cox proportional hazards models to assess hazard ratios (HRs) and 95% confidence intervals (CIs) for the association between TyG index and total and ischemic stroke risk. Models were adjusted for age, sex, body mass index (BMI), systolic blood pressure, diastolic blood pressure, total homocysteine, vitamin B12, estimated glomerular filtration rate (eGFR), MTHFR C677T, smoking status, alcohol drinking and treatment blood pressure. TyG index was assessed as a continuous and categorical variable: quartiles 1 to 4 and quartiles 1–2 versus quartiles 3–4. Cumulative event rates were estimated using the Kaplan–Meier method for outcomes that occurred in each of the quartile groups of TyG index. The joint effect of TyG index and age with stroke risk was further examined.

Additional stratified analyses were also conducted. To assess the modifying effects on the association between TyG and first stroke, we tested for interaction with potential covariates using interaction modeling. These covariates included age (< 60 versus ≥ 60 years), sex (male versus female), body mass index (< 24 versus ≥ 24 kg/m^2^), systolic blood pressure [< 165.3 (median) versus ≥ 165.3 mmHg], diastolic blood pressure [< 95.3 (median) versus ≥ 95.3 mmHg], estimated glomerular filtration rate [< 96.2 (median) versus ≥ 96.2 mL/min/1.73 m^2^], folate [< 7.3 (median) versus ≥ 7.3 ng/mL], homocysteine [< 12.4 (median) versus ≥ 12.4 μmol/L], vitamin B12 [< 371.0 (median) versus ≥ 371.0 pg/mL], MTHFR C677T polymorphisms (CC versus CT versus TT), smoking status (never smoker versus former smoker versus current smoker), and alcohol consumption (never drinker versus former drinker versus current drinker).

A 2-tailed P < 0.05 was considered to be statistically significant in all analyses. EmpowerStats (http://www.empowerstats.com) and R software, version 4.0.0 (http://www.R-project.org/), were used for all statistical analyses.

## Results

### Study participants and baseline characteristics

This analysis included 7569 adults with hypertension with no history of major cardiovascular disease at baseline. Table 1 illustrates the baseline characteristics of all participants stratified by median TyG index. As shown, those in the high TyG index group (TyG ≥ 8.8) tended to have higher levels of body mass index, systolic blood pressure, treatment systolic blood pressure, triglycerides, fasting glucose, eGFR and vitamin B12, and were more likely to be female. In addition, those in the high TyG index group had lower rates of smoking and alcohol consumption, and higher rates of a history of diabetes and hyperlipidemia.

**Table 1:** Baseline characteristics of participants by triglyceride-glucose index strata

	Triglyceride-glucose index < 8.8 (median)	Triglyceride-glucose index ≥ 8.8	*P*
N	3782	3787	
Female, No. (%)	2091 (55.3)	2487 (65.7)	< 0.001
Age, mean ± SD, year	59.5 (7.8)	59.4 (7.5)	0.614
Body mass index, mean ± SD, kg/m^2^	24.7 (3.4)	26.5 (3.5)	< 0.001
SBP, mean ± SD, mmHg	167.7 (21.0)	169.3 (21.1)	< 0.001
DBP, mean ± SD, mmHg	95.1 (12.0)	95.5 (12.2)	0.092
Treatment SBP, mean ± SD, mmHg	139.6 (11.6)	140.9 (11.7)	< 0.001
Treatment DBP, mean ± SD, mmHg	83.9 (7.6)	83.9 (7.7)	0.832
Laboratory results			
Triglycerides, median (IQR), mmol/L	1.1 (0.9, 1.3)	2.1 (1.7, 2.6)	< 0.001
Fasting glucose, median (IQR), mmol/L	5.4 (5.0, 5.9)	6.0 (5.4, 6.9)	< 0.001
eGFR, median (IQR), mL/min per 1.73 m^2^	94.8 (87.1, 101.0)	97.3 (89.3, 103.6)	< 0.001
Homocysteine, median (IQR), μmol/L	12.4 (10.4, 15.6)	12.4 (10.3, 15.7)	0.890
Folate, median (IQR), ng/mL	7.4 (5.3, 9.6)	7.2 (5.2, 9.4)	0.097
Vitamin B12, median (IQR), pg/mL	364.9 (309.0, 448.9)	378.1 (318.6, 469.5)	< 0.001
MTHFR C677T polymorphisms, No. (%)			0.068
CC	888 (23.5)	884 (23.3)	
CT	1928 (51.0)	1850 (48.9)	
TT	966 (25.5)	1053 (27.8)	
Cardiovascular risk factors, No. (%)			
Smoking status			< 0.001
Never smoker	2463 (65.1)	2798 (73.9)	
Former smoker	302 (8.0)	288 (7.6)	
Current smoker	1016 (26.9)	700 (18.5)	
Alcohol drinking			< 0.001
Never drinker	2522 (66.7)	2799 (73.9)	
Former drinker	257 (6.8)	230 (6.1)	
Current drinker	1000 (26.5)	758 (20.0)	
History of hyperlipidemia	78 (2.1)	145 (3.8)	< 0.001
History of diabetes	36 (1.0)	256 (6.8)	< 0.001

*SBP* systolic blood pressure, *DBP* diastolic blood pressure, *IQR* interquartile range, *eGFR* estimated glomerular filtration rate, *MTHFR* methylenetetrahydrofolate reductase

### Baseline TyG index associated with increased risk of first stroke

**Fig. 1:**
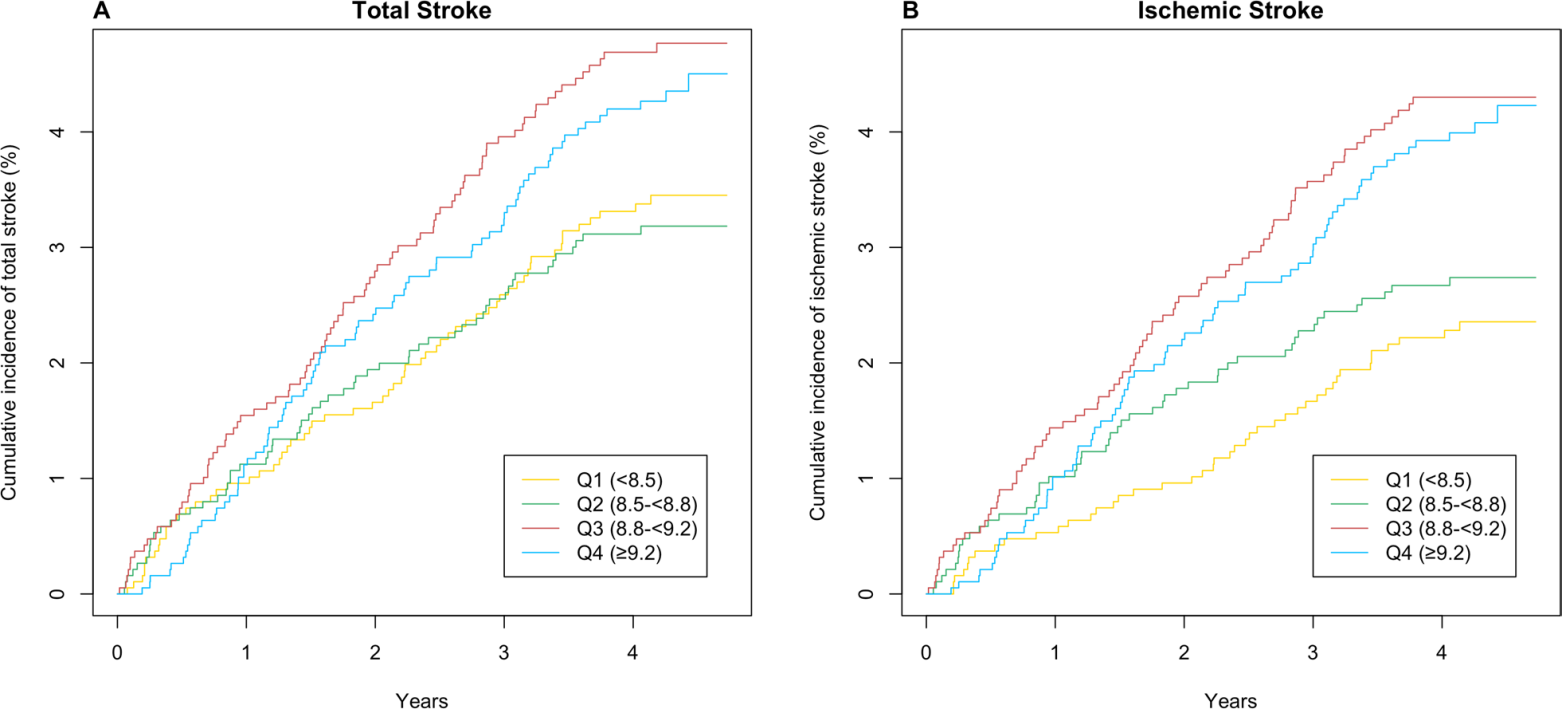
Kaplan–Meier curves of cumulative incidence of total stroke (**A**) and ischemic stroke (**B**) stratified by quartiles of baseline triglyceride-glucose index

Figure 1A and B show Kaplan–Meier curves for cumulative event rates for total stroke and ischemic stroke stratified by quartiles of baseline TyG index, respectively. The cumulative incidence of both total stroke and ischemic stroke tended to increase with increasing baseline TyG index and was more pronounced for TyG index ≥ 8.8. Consistently, compared with the reference group (Quartile 1: < 8.5), the hazard ratio of total stroke was 1.04 (95% CI 0.72–1.51) in Quartile 2 (8.5– < 8.8), 1.43 (95% CI 1.02–2.02) in Quartile 3 (8.8– < 9.2), and 1.45 (95% CI 1.01–2.08) in Quartile 4 (≥ 9.2), with a significant trend test (P = 0.013) after adjusting for covariables. When Quartiles 3 and 4 were further combined (Q3–Q4), the adjusted hazard ratio of total stroke was 1.41 (95% CI 1.10–1.81) compared with Q1–Q2 (Table 2).

**Table 2:** Association between baseline triglyceride-glucose index and risk of total and ischemic stroke

TyG index	N	Events (%)	Crude model		Adjusted model^*^	
			HR (95%CI)	*P*	HR (95%CI)	*P*
Total stroke						
Continuous	7569	288 (3.8)	1.30 (1.07, 1.59)	0.009	1.41 (1.14, 1.75)	0.002
Quartiles						
Q1 (< 8.5)	1892	63 (3.3)	ref		ref	
Q2 (8.5–8.8)	1890	58 (3.1)	0.93 (0.65, 1.33)	0.692	1.04 (0.72, 1.51)	0.822
Q3 (8.8–9.2)	1893	87 (4.6)	1.39 (1.00, 1.92)	0.047	1.43 (1.02, 2.02)	0.039
Q4 (≥ 9.2)	1894	80 (4.2)	1.27 (0.92, 1.77)	0.151	1.45 (1.01, 2.08)	0.042
P for trend				0.035		0.013
Categories						
Q1–Q2 (< 8.8)	3782	121 (3.2)	ref		ref	
Q3–Q4 (≥ 8.8)	3787	167 (4.4)	1.38 (1.09,1.74)	0.007	1.41(1.10,1.81)	0.007
P for trend				0.007		0.007
Ischemic stroke						
Continuous	7530	248 (3.3)	1.53 (1.24, 1.88)	< 0.001	1.66(1.33, 2.09)	< 0.001
Quartiles						
Q1 (< 8.5)	1882	43 (2.3)	ref		ref	
Q2 (8.5–8.8)	1883	51 (2.7)	1.20 (0.80, 1.80)	0.376	1.37(0.90, 2.08)	0.141
Q3 (8.8–9.2)	1883	79 (4.2)	1.86 (1.28, 2.69)	0.001	1.98(1.34, 2.93)	< 0.001
Q4 (≥ 9.2)	1882	75 (4.0)	1.76 (1.21, 2.56)	0.003	2.06(1.37, 3.09)	< 0.001
P for trend				< 0.001		< 0.001
Categories						
Q1–Q2 (< 8.8)	3765	94 (2.5)	ref		ref	
Q3–Q4 (≥ 8.8)	3765	154 (4.1)	1.64 (1.27, 2.12)	< 0.001	1.70 (1.29, 2.24)	< 0.001
P for trend				< 0.001		< 0.001

^*^Adjusted for age, sex, body mass index, systolic blood pressure, diastolic blood pressure, total homocysteine, vitamin B12, estimated glomerular filtration rate, MTHFR C677T, smoking status, alcohol drinking and treatment blood pressure

The TyG index showed the same trend in relation to ischemic stroke. When TyG index was analyzed as quartiles, compared with the reference group (Quartile 1: < 8.5), the hazard ratio of stroke was 1.37 (95% CI 0.90–2.08) in Quartile 2 (8.5– < 8.8), 1.98 (95% CI 1.34–2.93) in Quartile 3 (8.8– < 9.2), and 2.06 (95% CI 1.37–3.09) in Quartile 4 (≥ 9.2), with a significant trend test (P < 0.001) (Table 2).

### Subgroup analysis of the association between baseline TyG index and first stroke

The results of the stratified analysis showed that the relationship between baseline TyG index and first total stroke was modified by age and homocysteine (Additional file 1: Table S1). A baseline TyG index of more than 8.8 was associated with a significant 99% increased risk of stroke in those aged 60 years or older, whereas no significant findings were found in those younger than 60 years (P-interaction = 0.005). A significant interaction between TyG index and homocysteine for first total stroke was also observed (P-interaction = 0.010). Other variables, including sex, BMI, blood pressure, eGFR, folate, vitamin B12, MTHFR C677T polymorphisms, and smoking and alcohol consumption status did not modify the association between TyG index and stroke.

### The joint effect of baseline TyG index and age in relation to first stroke

**Fig. 2:**
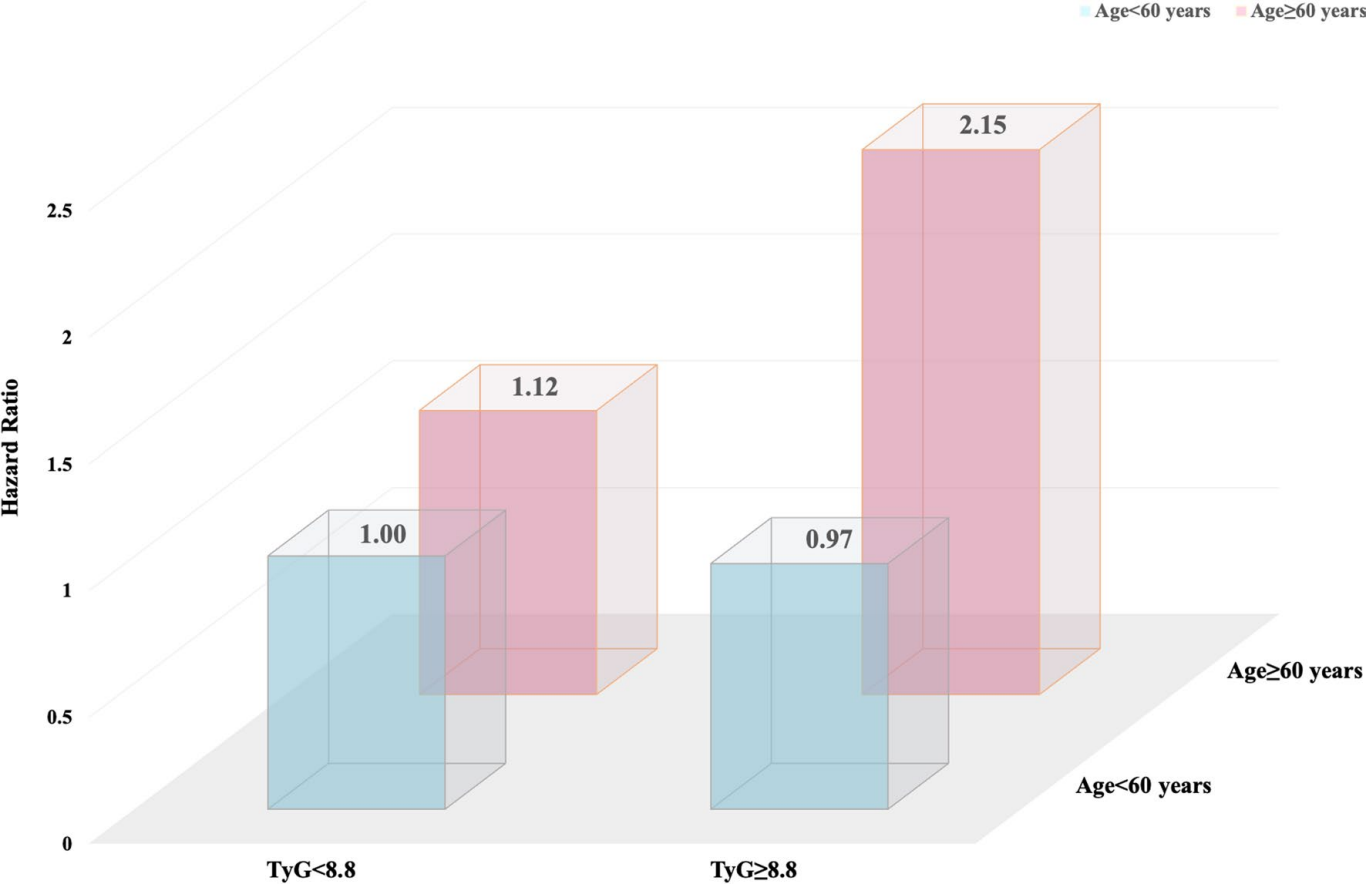
3D display of total stroke hazard ratios by baseline triglyceride-glucose index and age

Figure 2 shows a 3D display of total stroke hazard ratios by baseline TyG index and age. As shown in the figure, a higher TyG index was associated with a higher risk of stroke only in the elderly group (aged 60 years or greater). Table 3 illustrates the joint effect of TyG index and age on the risk of first stroke. Using TyG index < 8.8 and age < 60 years as reference, a higher TyG index was significantly associated with a higher stroke risk only for those aged 60 or greater (HR: 2.15, 95%CI 1.50, 3.07, P < 0.001).

**Table 3:** The joint effect of baseline triglyceride-glucose index and age on the risk of total stroke

Triglyceride-glucose index	Age, year	N	Events (%)	HR (95%CI)	*P*
< 8.8	< 60	2050	61 (3.0)	ref	
≥ 8.8	< 60	2062	61 (3.0)	0.97 (0.67, 1.40)	0.864
< 8.8	≥ 60	1732	60 (3.5)	1.12 (0.75, 1.66)	0.587
≥ 8.8	≥ 60	1725	106 (6.1)	2.15 (1.50, 3.07)	< 0.001
*P* for trend					< 0.001

Adjusted for sex, body mass index, systolic blood pressure, diastolic blood pressure, total homocysteine, vitamin B12, estimated glomerular filtration rate, MTHFR C677T, smoking status, alcohol drinking and treatment blood pressure

### The joint effect of baseline TyG index and homocysteine in relation to first stroke

Additional file 1: Table S2 demonstrates the joint effect of baseline TyG index and homocysteine on the risk of total stroke. Those with a TyG index < 8.8 and a homocysteine level < 12.4 μmol/L were the reference group. An independent elevation in homocysteine was associated with a 66% increased risk of stroke (HR: 1.76, 95%CI 1.15, 2.69, P = 0.009) and an independent elevation in TyG index was associated with a stroke hazard of 2.14 (95%CI 1.41, 3.24, P < 0.001). The risk of stroke was further increased for the group with elevated TyG combined with elevated homocysteine, with a stroke hazard of 1.95 (95%CI 1.28, 2.95, P = 0.002) and a significant trend test (P = 0.003).

## Discussion

The present study is a prospective cohort study with a median follow-up time of 4.5 years. To our knowledge, this study is the first to explore the predictive value of baseline TyG index levels on first stroke risk in a hypertensive population, and to identify associated modifiers. In this study, a baseline TyG index greater than or equal to 8.8 was found to be associated with a significant 41% increased risk of total stroke and a 70% increased risk of ischemic stroke in a Chinese population with hypertension. Further, we found that age significantly influenced the association of TyG with stroke, where a higher TyG index was significantly associated with a higher risk of stroke only in people aged 60 years and older (HR: 2.15; 95% CI 1.50–3.07).

In 2008, Simental-Mendía et al. first proposed TyG index, a composite index consisting of triglyceride and fasting glucose levels [3]. As a surrogate marker of insulin resistance (IR), TyG index is reliable, readily available and of low-cost, is proven to have higher sensitivity and specificity than traditional measures, and is therefore, better suited for clinical practice and large epidemiological studies [4, 10]. In 2016, the first association between TyG index and cardiovascular disease was confirmed [11]. Subsequently, there has been a series of evidence to show that TyG index is associated with a variety of cardiovascular diseases such as heart failure [12], arterial stiffness [13], and acute coronary syndromes [14]. In recent years, studies have demonstrated the value of TyG index in stroke prevention. A cross-sectional study based on a population in northern China found a significant, positive association between TyG index and ischemic stroke [6]. Wang et al. found that baseline TyG index was significantly associated with an increased risk of first stroke and ischemic stroke among a community population [7]. A meta-analysis that pooled eight cohort studies showed similar results, with the researchers concluding that the association between TyG index and stroke was independent of sex, age, and diabetes status [15]. However, evidence of an association within the hypertensive population is limited. As a population at high risk for stroke [16], understanding this association is of great significance.

This study is the first to confirm that a higher baseline TyG index is associated with an increased risk of total stroke and ischemic stroke in a hypertensive population, and these results are generally consistent with the results of previous studies in non-hypertensive populations. More importantly, this study found for the first time that age significantly affects the stroke predictive value of TyG index: those patients aged 60 years or more with a higher TyG index had up to 2.15 times higher stroke risk compared to those under age 60. This may be related to long-term accumulated vascular damage in the older hypertensive population, highlighting the importance of large screenings in elderly populations (aged 60 years or older) with hypertension. In addition, this study additionally found a synergistic effect of homocysteine with TyG index. Previous evidence suggests that homocysteine induces insulin resistance and a diabetic phenotype by acting on the pro-insulin receptor through protein cysteine-homocysteinylation (C-Hcy) [17]. The Framingham Offspring Study also found an association between hyperhomocysteinemia and an increased risk of cardiovascular disease (CVD) related to insulin resistance [18]. As independent risk factors for stroke, these findings all help to further refine the identification of people at high risk for stroke.

The exact mechanism of the TyG-stroke relationship is unclear, but evidence indicates that the TyG index is associated with subclinical atherosclerosis [19–22]: a higher TyG index is associated with a 56% increased risk of abnormal mean common carotid artery intima-media thickness (cIMT) [21] and a 64.8% increased risk of coronary plaque progression [22], and subsequently increased stroke risk. As a novel clinical marker for insulin resistance, the underlying mechanism may be related to the contribution of IR and IR-related metabolic diseases to the development of atherosclerosis and subsequent stroke. Studies have demonstrated that IR may contribute to stroke onset and progression through multiple pathways: platelet activation; aggregation [23–25]; endothelial dysfunction [26]; smooth muscle cell dysfunction [27]; or overactivation of the renin-angiotensin system [28], among others.

As a simple, inexpensive and effective alternative to IR, the TyG index is expected to provide more value in the field of stroke prevention in the future, especially as a stroke screening tool for large studies. Currently, stroke remains a serious global public health problem [29], and identifying valid and actionable indicators for early identification is a global health priority. Unlike traditional IR testing methods, the TyG index can be obtained through blood tests and simple calculations. It is also cheaper and easier to perform than IR testing, making it an important practical tool especially among developing countries and regions. In addition, studies have shown that insulin resistance leads to increased sympathetic nervous system activity, over-activation of the renin-angiotensin system, and increased renal sodium retention, which, in concert with hypertension, contributes to the development and progression of cardiovascular disease such as stroke [30, 31]. Therefore, determining the relationship between IR and stroke in a hypertensive population has important clinical and public health implications.

There are still some limitations of this study. First, TyG index was calculated from a single blood test result at baseline, and the effect of fluctuations in blood glucose and triglyceride levels over time cannot be excluded. Second, although this study found that a TyG index above 8.8 was associated with a significantly higher risk of stroke, future studies are needed to determine the optimal cut point value. Third, other traditional IR indicators were not measured at baseline in this study, and their value for stroke prevention cannot be compared with that of the TyG index. Finally, the data for this study were from the Lianyungang center of the CSPPT study. The extrapolation of our findings may be restricted, and more studies in Chinese and foreign populations are needed to validate our results.

## Conclusions

This study within a Chinese population with hypertension validated that baseline TyG index was associated with a significantly increased risk of stroke and further identified a modifying effect of age and homocysteine. As a reliable, low-cost, alternative marker of IR that can be obtained by blood tests and simple calculations, the TyG index is suitable for large studies and screening purposes, and is expected to play an important role in the field of stroke prevention in the future.

### Supplementary Information

**Additional file 1: Figure S1.** Flowchart of the study. **Table S1.** Subgroup analyses on the association between triglyceride-glucose index and risk of total stroke. **Table S2.** The joint effect of baseline triglyceride-glucose index and homocysteine on the risk of total stroke. **Table S3.** Comparison of baseline characteristics between the included and excluded populations.

## Data Availability

Data, analytical methods and research materials supporting the results of this study may be reasonably requested from the corresponding author after the application has been submitted and formally reviewed and approved by the Ethics Committee of the Institute of Biomedical Research, Anhui Medical University.

## Abbreviations

CI: Confidence interval
cIMT: Common carotid artery intima-media thickness
CKD-EPI: Chronic Kidney Disease Epidemiology Collaboration
CSPPT: China Stroke Primary Prevention Trial
eGFR: Estimated glomerular filtration rate
HR: Hazard ratio
IR: Insulin resistance
MTHFR: Methylenetetrahydrofolate reductase
STROBE: Strengthening the Reporting of Observational Studies in Epidemiology
TyG: Triglyceride-glucose
