# Supplementary material for: Triglyceride-glucose index is associated with a higher risk of stroke in a hypertensive population
Source: Cardiovasc Diabetol. 2023 Dec 13;22:346. doi: 10.1186/s12933-023-02082-1 (PMC10720217; doi:10.1186/s12933-023-02082-1)
Supplement: Supplementary file 1 — Additional file 1: Figure S1. Flowchart of the study. Table S1. Subgroup analyses on the association between triglyceride-glucose index and risk of total stroke. Table S2. The joint effect of baseline triglyceride-glucose index and homocysteine on the risk of total stroke. Table S3. Comparison of baseline characteristics between the included and excluded populations. [file 12933_2023_2082_MOESM1_ESM.pdf]

Supplementary Materials

Figure S1. Flowchart of the study

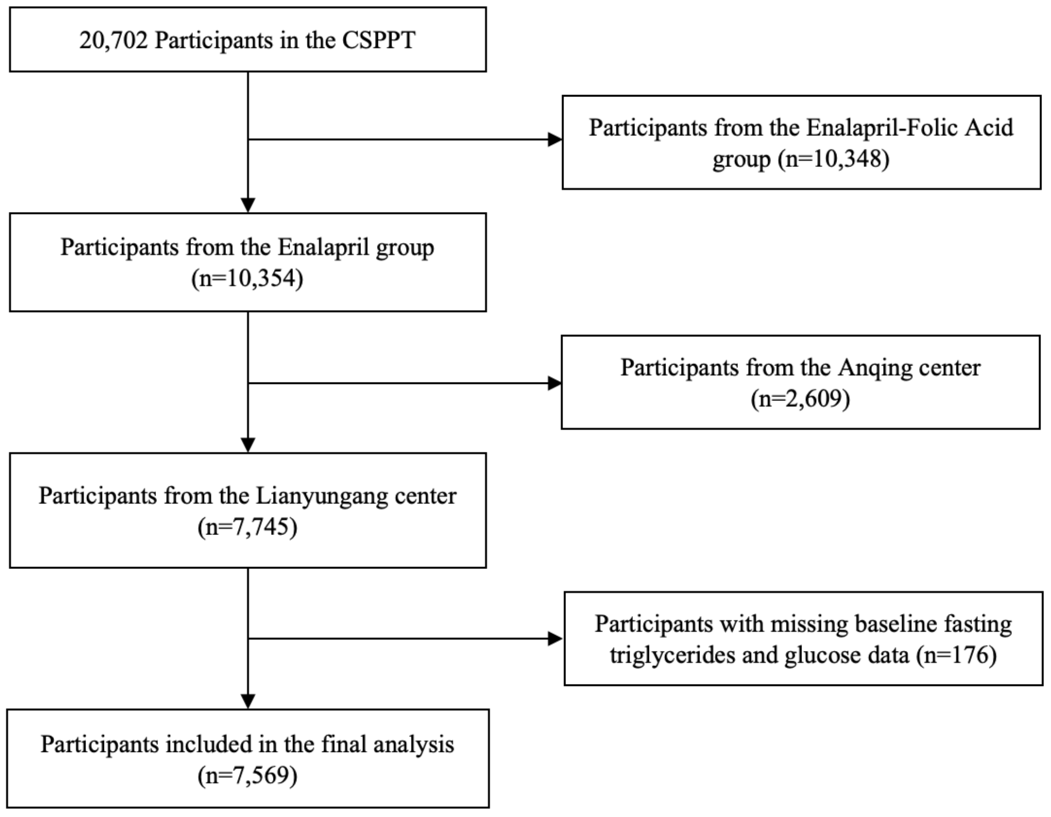

Table S1. Subgroup analyses on the association between triglyceride-glucose index and risk of total stroke*

Subgroup	TyG Index Q1-Q2 (<8.8)		TyG Index Q3-Q4 (≥8.8)		HR (95%CI)	P for interaction
	N	Events (%)	N	Events (%)		
**Age, y**						0.005
**<60 (median)**	2050	61(3.0)	2062	61(3.0)	0.91(0.61,1.33)	
**≥60**	1732	60(3.5)	1725	106(6.1)	1.99(1.42,2.79)	
**Sex**						0.327
**Male**	1691	75(4.4)	1300	72(5.5)	1.34(0.94,1.90)	
**Female**	2091	46(2.2)	2487	95(3.8)	1.47(1.01,2.13)	
**Body Mass Index, kg/m^2**						0.221
**<24**	1636	53(3.2)	891	47(5.3)	1.77(1.17,2.66)	
**≥24**	2145	68(3.2)	2896	120(4.1)	1.24(0.91,1.69)	
**Systolic blood pressure, mmHg**						0.687
**<165.3(median)**	1930	38(2.0)	1808	43(2.4)	1.19(0.75,1.90)	
**≥165.3**	1852	83(4.5)	1979	124(6.3)	1.55(1.15,2.09)	
**Diastolic blood pressure, mmHg**						0.436
**<95.3(median)**	1882	45(2.4)	1870	68(3.6)	1.72(1.15,2.58)	
**≥95.3**	1900	76(4.0)	1917	99(5.2)	1.28(0.93,1.77)	
**eGFR, mL/min per 1.73 m^2**						0.724
**<96.2(median)**	2079	76(3.7)	1705	92(5.4)	1.59(1.15,2.19)	
**≥96.2**	1702	45(2.6)	2082	75(3.6)	1.20(0.81,1.77)	
**Folate, ng/mL**						0.140
**<7.3(median)**	1841	70(3.8)	1900	85(4.5)	1.24(0.88,1.73)	
**≥7.3**	1890	48(2.5)	1853	79(4.3)	1.69(1.15,2.47)	
**Homocysteine, μmol/L**						0.010
**<12.4(median)**	1892	35(1.8)	1887	74(3.9)	2.05(1.33,3.17)	
**≥12.4**	1888	86(4.6)	1895	93(4.9)	1.11(0.81,1.51)	
**Vitamin B12, pg/mL**						0.885
**<371(median)**	1960	63(3.2)	1782	85(4.8)	1.41(1.00,1.99)	
**≥371**	1771	55(3.1)	1971	79(4.0)	1.46(1.01,2.11)	
**MTHFR C677T polymorphisms**						0.134
CC	888	31(3.5)	884	41(4.6)	1.47(0.89,2.41)	
CT	1928	49(2.5)	1850	81(4.4)	1.78(1.21,2.63)	
TT	966	41(4.2)	1053	45(4.3)	0.95(0.60,1.50)	
**Smoking status**						0.318
Never smoker	2463	60(2.4)	2798	109(3.9)	1.48(1.06,2.06)	
Former smoker	302	10(3.3)	288	20(6.9)	1.37(0.61,3.11)	
Current smoker	1016	51(5.0)	700	38(5.4)	1.31(0.83,2.07)	
**Alcohol drinking**						0.195
Never drinker	2522	63(2.5)	2799	116(4.1)	1.61(1.16,2.24)	

Former drinker	257	7(2.7)	230	13(5.7)	1.22(0.45,3.35)
Current drinker	1000	51(5.1)	758	38(5.0)	1.13(0.72,1.78)

*Adjusted, if not stratified, for age, sex, body mass index, systolic blood pressure, diastolic blood pressure, total homocysteine, vitamin B12, estimated glomerular filtration rate, MTHFR C677T, smoking status, alcohol drinking and treatment blood pressure.

Abbreviations: eGFR, estimated glomerular filtration rate.

Table S2. The joint effect of baseline triglyceride-glucose index and homocysteine on the risk of total stroke *

TyG index	Hcy, µmol/L	N	Events (%)	HR (95%CI)	*P*
**<8.8**	**<12.4**	1892	35 (1.8)	ref	
**<8.8**	**≥12.4**	1888	86 (4.6)	1.76 (1.15, 2.69)	0.009
**≥8.8**	**<12.4**	1887	74 (3.9)	2.14 (1.41, 3.24)	<0.001
**≥8.8**	**≥12.4**	1895	93 (4.9)	1.95 (1.28, 2.95)	0.002
***P* for trend**					0.003

*Adjusted for age, sex, body mass index, systolic blood pressure, diastolic blood pressure, vitamin B12, estimated glomerular filtration rate, MTHFR C677T, smoking status, alcohol drinking and treatment blood pressure

Table S3. Comparison of baseline characteristics between the included and excluded populations

	Included Population	Excluded Population	*P*
**N**	7569	13133	
**Female, No. (%)**	4578 (60.5)	7627 (58.1)	<0.001
**Age, mean ± SD, y**	59.4 (7.6)	60.3 (7.5)	<0.001
**Body mass index, mean ± SD, kg/m^2**	25.6 (3.5)	24.6 (3.7)	<0.001
**SBP, mean ± SD, mmHg**	168.5 (21.1)	166.0 (19.9)	<0.001
**DBP, mean ± SD, mmHg**	95.3 (12.1)	93.4 (11.8)	<0.001
**Treatment SBP, mean ± SD, mmHg**	140.2 (11.6)	139.6 (11.3)	<0.001
**Treatment DBP, mean ± SD, mmHg**	83.9 (7.7)	82.6 (7.6)	<0.001
**Laboratory results**			
eGFR, median (IQR), mL/min per 1.73 m^2	96.2 (88.1, 102.3)	95.1 (87.0, 101.7)	<0.001
Homocysteine, median (IQR), μmol/L	12.4 (10.3, 15.6)	12.6 (10.6, 15.4)	0.217
Folate, median (IQR), ng/mL	7.3 (5.2, 9.5)	8.6 (5.9, 11.1)	<0.001
Vitamin B12, median (IQR), pg/mL	371.0 (313.4, 459.8)	384.6 (315.9, 486.1)	<0.001
**MTHFR C677T polymorphisms, No. (%)**			<0.001
CC	1772 (23.4)	3880 (29.5)	
CT	3778 (49.9)	6398 (48.7)	
TT	2019 (26.7)	2885 (21.7)	
**Cardiovascular risk factors, No. (%)**			
Smoking status			0.078
Never smoker	5261 (69.5)	8993 (68.5)	
Former smoker	590 (7.8)	980 (7.5)	
Current smoker	1716 (22.7)	3153 (24.0)	
Alcohol drinking			0.002
Never drinker	5321 (70.3)	8950 (68.2)	
Former drinker	487 (6.4)	972 (7.4)	
Current drinker	1758 (23.2)	3202 (24.4)	
History of hyperlipidemia	223 (2.9)	339 (2.6)	0.120
History of diabetes	292 (3.9)	360 (2.7)	<0.001

Abbreviations: SBP, systolic blood pressure; DBP, diastolic blood pressure; IQR, interquartile range; eGFR, estimated glomerular filtration rate; MTHFR, methylenetetrahydrofolate reductase.
